# The Angular Interval between the Direction of Progression and Body Orientation in Normal, Alcohol- and Cocaine Treated Fruit Flies

**DOI:** 10.1371/journal.pone.0076257

**Published:** 2013-10-16

**Authors:** Anna Gakamsky, Efrat Oron, Dan Valente, Partha P. Mitra, Daniel Segal, Yoav Benjamini, Ilan Golani

**Affiliations:** 1 Department of Zoology, Tel Aviv University, Tel Aviv, Israel; 2 Cold Spring Harbor Laboratory, Cold Spring Harbor, New York, United States of America; 3 Molecular Microbiology and Biotechnology, Tel Aviv University, Tel Aviv, Israel; 4 Department of Statistics and OR, Tel Aviv University, Tel Aviv, Israel; 5 Sagol School of Neuroscience, Tel Aviv University, Tel Aviv, Israel; Tulane University Medical School, United States of America

## Abstract

In this study we characterize the coordination between the direction a fruit-fly walks and the direction it faces, as well as offer a methodology for isolating and validating key variables with which we phenotype fly locomotor behavior. Our fundamental finding is that the angular interval between the direction a fly walks and the direction it faces is actively managed in intact animals and modulated in a patterned way with drugs. This interval is small in intact flies, larger with alcohol and much larger with cocaine. The dynamics of this interval generates six coordinative modes that flow smoothly into each other. Under alcohol and much more so under cocaine, straight path modes dwindle and modes involving rotation proliferate. To obtain these results we perform high content analysis of video-tracked open field locomotor behavior. Presently there is a gap between the quality of descriptions of insect behaviors that unfold in circumscribed situations, and descriptions that unfold in extended time and space. While the first describe the coordination between low-level kinematic variables, the second quantify cumulative measures and subjectively defined behavior patterns. Here we reduce this gap by phenotyping extended locomotor behavior in terms of the coordination between low-level kinematic variables, which we quantify, combining into a single field two disparate fields, that of high content phenotyping and that of locomotor coordination. This will allow the study of the genes/brain/locomotor coordination interface in genetically engineered and pharmacologically manipulated animal models of human diseases.

## Introduction

Representations of insect movement, indispensible for studying the interface between genes brain and behavior, have suffered for several decades from a gap: on the one hand, neuroethological studies of insect behavior involving well-defined and circumscribed situations such as prey capture or gait analysis typically include state-of-the-art low-level descriptions consisting of dynamic representations of kinematic measures. On the other hand, studies in behavior genetics and behavioral pharmacology involving extended Open Field behavior typically use cumulative measures, expert-defined behavior patterns based on subjective decisions, and selected drawings of path traces.

Progress in video-tracking technology now allows the characterization of the animals' path. Even with the simplification of an animal as a moving point, much has been learned about locomotor behavior of vertebrates [1]–[6] and invertebrates [7]–[14]. With the capability to also track the orientation of the animal's body [15]–[19] one might have expected a shift toward a phenotyping based on quantifiable dynamics of coordination between translation and body orientation, yet, the obtained high quality data are often used to either compare, as before, cumulatively assembled data or else reinstate the patterns of classical ethology. These “black boxes” are a mixed blessing: they are useful for scoring the behavior of closely related phenotypes but are too high level for comparing apparently dissimilar behavioral preparations. Furthermore, they arguably lack sufficient content for studying coordination [20]. Few studies do start, however, with the underlying dynamics of kinematic variables and then proceed to show that the animal's behavioral repertoire is generated by these dynamics. This has been done, for example, for carnivore [21] and rodent [22] locomotor behavior, for rodent gait [23], and for worm [24]–[27] and fly larva [28], [29] locomotor behavior. The present study similarly provides a bottom-up alternative whereby low-level kinematic variables – translation and body-orientation-in-the-horizontal-plane – are used to construct higher level constructs in fruit fly locomotor behavior. We characterize the dynamics of the angular interval between the direction the animal walks and the direction it faces (hence *angular interval*) in intact flies and provide support that this interval is actively managed by demonstrating it can be modulated in a patterned way with drugs. Our analysis reveals that administration of alcohol increases the *angular interval*, and administration of cocaine increases it much further. Alcohol, and much more so cocaine, also reduce the proportion of walking on straight paths and increase the prevalence of modes involving rotation. Seemingly bizarre, formerly inexplicable behaviors performed with alcohol and cocaine, like walking sideways or backwards, become almost inevitable manifestations of behaviors involving a large angular interval. Most important, our results establish the 3 low-level variables, progression, facing. and the angular difference between their respective directions, as key actively managed variables, and 6 higher level modes generated by the dynamics of the angular interval such as Fixed-front-on-Straight-Path, Rotation-on-Straight-Path, Fixated-Front-on-Curved-Path, and Rotation-on-Curved-Path as fundamental constructs whose quantification discriminates between treatments, validates our descriptive model and demonstrates its usefulness for phenotyping. The present study combines two disparate fields, that of high content phenotyping and that of locomotor coordination, into a single field of study.

## Materials and Methods

The first part of the methods section is dedicated to the application of density functions that establish intrinsic cutoff points between segments and episodes. The insistence on intrinsic cutoff points and measures that are customized to fit as closely as possible the actual data (as opposed to using intuitive or even “reasonable” but arbitrary cutoff points) is essential for obtaining results that have the potential of being replicable across laboratories [20], a fundamental prerequisite for a science of behavior.

### Animals

*Drosophila* cultures were maintained at 24°C on a standard cornmeal/molasses medium in 12-h light: 12-h dark cycle at 60% humidity. The wild-type laboratory strain Canton-S (CS) was used. 3 groups, each having 8 three-day-old male flies were videotaped. To reduce a potential source of variation only males, suspected to show higher levels of activity [30], were used.

### Experimental setup

**Figure 1:**
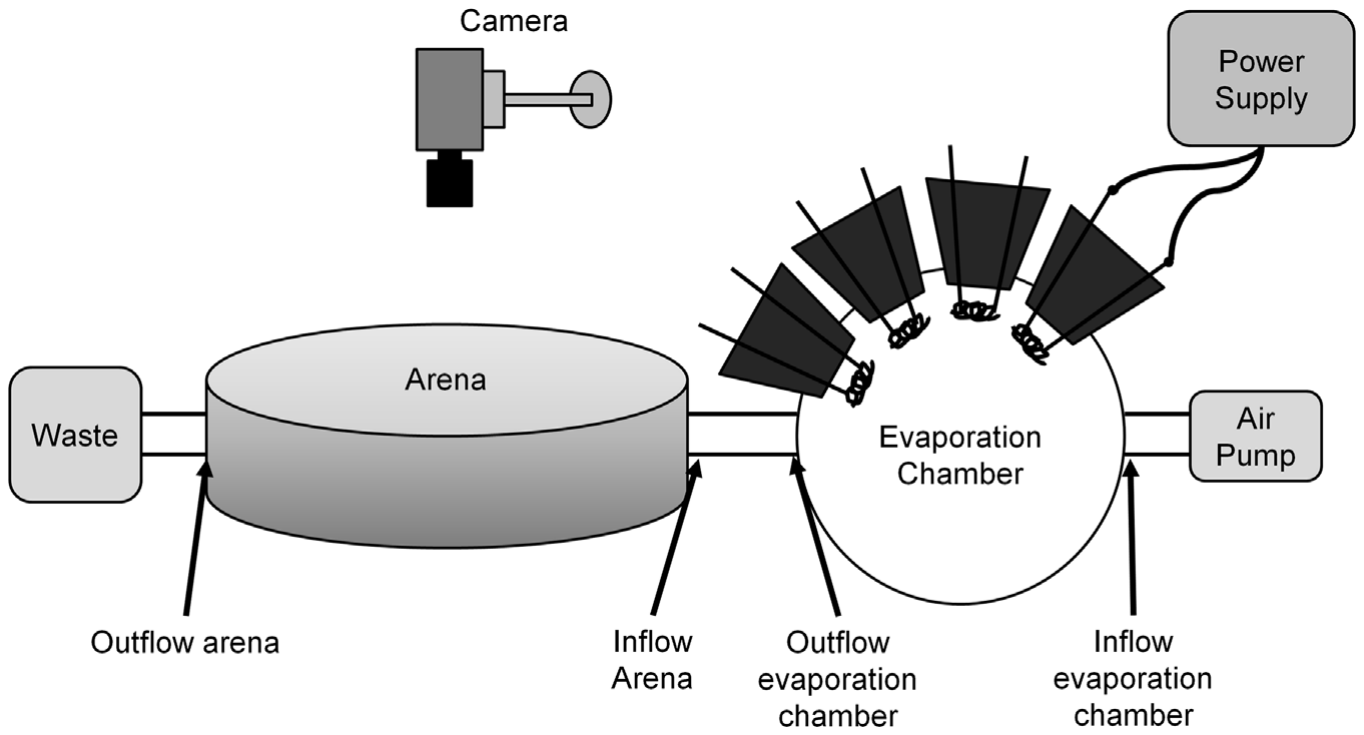
Schematic drawing of the arena and drug administration apparatus used to quantify spontaneous locomotor activity of a single fly. Cocaine, ethanol, or air is pumped into the arena from the evaporation chamber.

All experiments were performed during the 12 hrs light period, on one fly at a time. Neither food nor water was supplied to the fly during the entire experiment. The experimental setup for observing and tracking the flies was a 15 cm diameter circular arena with 0.7 cm height, which was illuminated from above by a 40 W bulb (Figure 1). A thin, transparent plastic ceiling was placed over the arena so that the fly did not escape during testing.

Two small openings in the arena wall allowed air flow and introduction of volatilized drugs – cocaine or alcohol – into the arena during the experiment. The drug volatilizing apparatus was connected to the arena by a short pipe. Cocaine was volatilized in a transparent, perspex chamber consisting of four volatilizing units. Each unit consisted of a nichrome wire connected to copper leads that were passed through a neoprene stopper and connected to a low voltage/high current regulated power supply [31]. The volatilizing chamber was connected both to the arena and to an air pump securing air flow through the volatilizing chamber into the arena. Free base Cocaine (150 ug) was volatilized from the nichrome filaments as follows: Free base cocaine dissolved in ethanol was applied to the filament and ethanol was allowed to evaporate. Evaporation of the cocaine was done using a low voltage/high current regulated power supply by applying a voltage sufficient to heat the filament to 200°C within 5 sec [31]).

### Drug administration

The fly was transferred to the arena and allowed to habituate for 1 hour. Then its behavior was recorded for 1 hour. In the treated flies a pre-determined amount of cocaine or alcohol was streamed into the arena at a constant rate over a specified period of time. Ewing [32] and later Connoly [30] showed that different populations of flies differed in their reactivity to environmental stimuli but not in spontaneous activity. Therefore, we performed the experiment over an extended period of time. In this way drug treatment was given without disturbing the fly with the presence of other flies or with a novel environment yielding spontaneous, rather than reactive, behavior [8], [30], [32]. Following exposure to cocaine, fly behavior was recorded for an additional 2 hrs. Based on preliminary experiments, this time was found to be sufficient for the fly to be influenced by the drug and then to regain normal behavior – be it with cocaine or with alcohol. The behavior of all drug-treated flies has been analyzed from the moment the drug started to be streamed into the arena chamber until complete fly sedation. The behavior following sedation was not analyzed in the present study. Videotaping recovery from sedation was necessary in order to ascertain that the dose used was not lethal and the fly consequently recovered normal behavior. Cocaine-treated activity included in average, from start to full sedation, 3 minutes per fly and alcohol 48 minutes. Normal fly sessions included 167 minutes each.

### Determination of the fly's center and of body orientation

Video acquisition was performed at 25 Hertz (40 ms time step) at a resolution of 720×560 pixels using a CCD camera placed above the arena. The spatial resolution was 1.5 pixel/mm. In this study we use an adaptation of FTrack, a Matlab toolbox for trajectory tracking and analysis, to record both a fly's position and its orientation. [15]. FTrack determines the location of the centroid and the orientation of the longitudinal axis of the fly's body. To do this, FTrack creates a background, subtracts it from the current frame, and squares each resulting pixel to increase the signal to noise ratio. Then, the darkest pixel in this image is found and the “center of mass” (center of intensity) of a subset of pixels around this point is calculated. This center of intensity is used as the object's location (FTrack v0.9, User's Manual [15]).

Body axis position is calculated by Principal Component Analysis on the above subset of pixels. Since a fly is typically longer than it is wide, the component with the largest variance is used to calculate the body axis angle α_1_ (FTrack v0.9, User's Manual, [15]. FTrack provides this angle as well as its conjugated angle α_2_  =  α_1_ + π, which defines the same axis. The raw data are then corrected for tilt and rotation of the camera [15] and data corresponding to the fly's presence on the wall and jumps are excluded. These data are excluded for two reasons. First, the fly tends to be vertical on the wall and does not always move parallel to the plane of the open field, and second, movement on the wall is physically constrained and perceptually different. For this study, we are only interested in free, unconstrained movement on a horizontal surface and these properties are violated at the boundaries of the arena.

FTrack is not able to unambiguously define the head of the fly. To determine which of the two conjugate orientation angles, α_1_ and α_2_, is the correct angle to be used when relating the fly's body orientation to its direction of progression, we select, for each wall-to-wall segment, a frame with a high speed of progression and use it as a reference frame: in that frame the fly's head faces the direction of progression. Orientation angles for the rest of the segment are determined by minimizing frame-to-frame change in orientation (selecting the smaller of the two conjugate orientation angles, α_1_ and α_2)_, under the observation that large rotational speeds are highly unlikely to occur in a single frame (40 ms). In other words, flies do not perform a 180° body rotation in the course of 40 ms – they do not shift in the course of a single frame from walking forward in one direction to walking forward in the opposite direction. Reversion of the velocity vector's direction in the course of a single frame implies therefore that the fly walked backwards. This algorithm distinguishes head from tail and captures all backward progression episodes.

### Data smoothing and velocity determination

The coordinates of the fly's center (*X*_c_ and *Y*_c_) and body orientation angle α_b_ were smoothed through a combination of LOWESS and Repeated Running Median procedures [33]. This produces reliable estimates of the numerical derivatives of the raw data. Derivatives of the centre coordinates, 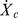 and 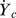, allow calculation of the magnitude *V*_c_ (speed) and direction α_v_ of the instantaneous velocity vector:
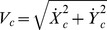


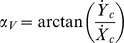



The derivative of the body orientation angle represents the angular velocity of body rotation, ω_b_. Smoothing of the orientation angle α_vc_ by the above procedure provides the angular velocity of rotation of the velocity vector, ω_v_.

### Determination of threshold values for movement segmentation

#### Progression vs. non-progression segments

**Figure 2:**
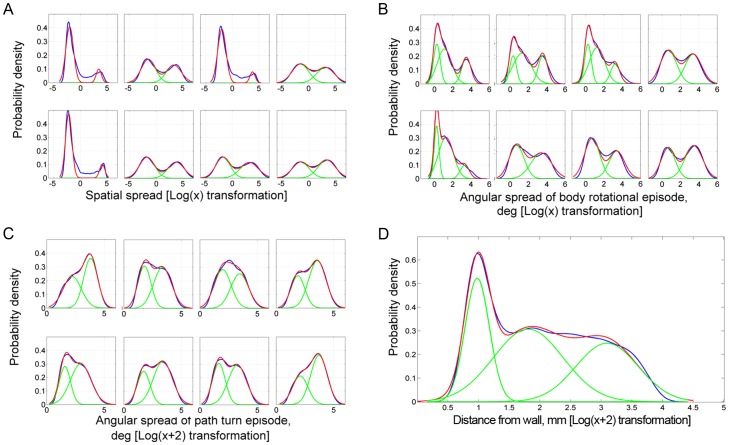
Density plots of spatial and angular parameters. Blue line – empirical distribution, green line – the Gaussians established by the EM algorithm, red line – the algebraic summation of the two Gaussians. The threshold value is provided by the x-value at the intersection point between the Gaussians.A) Spatial spread values for progression segments extending between two successive arrests; B) Angular spread values for segments extending between 2 successive arrests in *rotation of body axis*. The left peak of the density plot has an asymmetrical shape, which is in some cases better fitted with two Gaussians. We considered this peak as representing fluctuations in orientation caused by both noise in the detection system and in real small body movements (body wobble). Since we were interested in identification of segments with pronounced body rotations, an intersection point between the two rightmost Gaussians was accepted as the threshold for angular spread above which a segment was counted as a body rotation;. C) Angular spread values for segments extending between 2 successive arrests of the velocity vector (intervals in which the fly's path direction is stationary); D) Distances from the wall for all data points belonging to progression segments. The leftmost Gaussian corresponds to the wall zone, the middle to the near wall zone, and the rightmost corresponds to the central zone. Data were pooled from 8 intact flies. The intersection point between the middle and the rightmost peaks (10 mm) was chosen as a boundary defining the central zone of the arena.

For each fly, segments of putative progression were selected from the entire location time series as those bounded by two successive points with V_c_ = 0 (arrests). We define the spatial spread as the maximal distance between any two data points belonging to the examined inter-arrests segment, and this was calculated for each of the inter-arrests segments. A per-fly density function of the spatial spread values were fit with Gaussians (Figure 2). The intersection points between the Gaussians were averaged across flies, resulting in a threshold value of 4.7 mm (approximately one fly's body length). Segments with spatial spreads above this threshold were counted as a movement segment.

#### Body rotation vs. fixed body orientation

For each fly, segments of putative body rotation were selected from the time series of angular velocity ω_b_, as those bounded by two successive points with ω_b_ = 0. As with the computation of spatial spread, angular spread – the maximal angular distance between any two angular values belonging to the examined inter-arrests segment – was defined and calculated within each of the above segments. Per-fly density plots of these values were then fitted with Gaussians (Figure 2B). An intersection point between the Gaussians was accepted as the threshold for angular spread above which a segment was counted as a body rotation segment. Averaged across flies, the obtained value was 12°.

#### Curved path vs straight path

For each fly, we first selected segments of progression within the inner part of the arena (Figure 2D). Next, for these segments, we selected episodes of a putative change in path curvature by examining the corresponding time series of angular velocity, ω_v_, enclosed between two consecutive points with ω_v_  = 0. Angular spread within each of the above segments was calculated and per-fly density plots of these values were fitted with Gaussians (Figure 2C). The average value of the intersection points between Gaussians established a threshold value of 13°. This value was used to distinguish straight from curved paths.

### Partitioning of arena to spatial zones

Partitioning of the arena into spatial zones was performed on the basis of the spatial distribution of movement segments' data. For each point in a progression segment, the radial position *R_i_* and the distance from the wall *d_i_* were calculated as
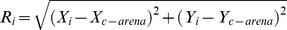


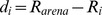
where *X_c-arena_, Y_c-arena_* and *R_arena_* are coordinates of the centre and radius of the arena. Density plots of values *d_i_* were fit with Gaussians (Figure 2D). Three zones were defined: the wall zone, the near wall zone and the central one. Culling out and then studying only the behavior in the open space, away from walls, is more likely to highlight endogenous constraints, imposed on the fly's trajectory by the CNS.

### Partitioning of cocaine response into stages

**Figure 3:**
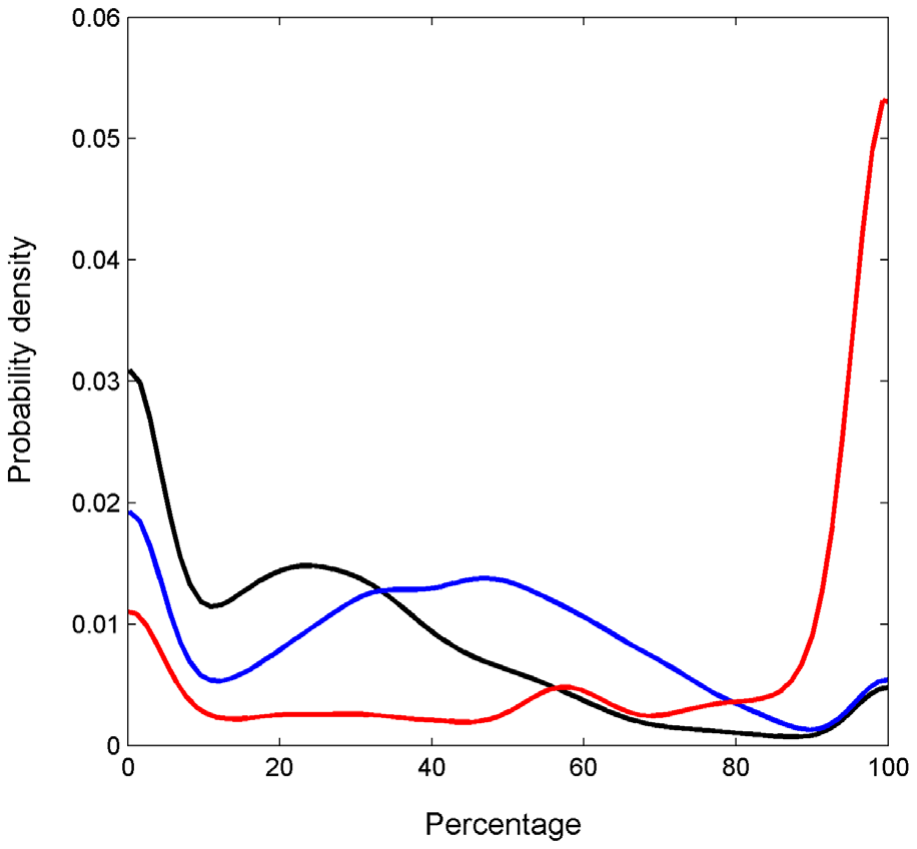
Cumulative percentages of three rotational modes within movement segments. Black – untreated flies, blue – alcohol treated flies, red – cocaine treated flies.

**Figure 4:**
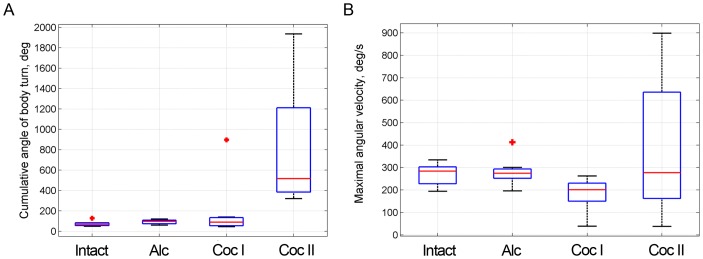
Box plot summaries of rotations of intact and drug-treated flies. A) Cumulative angle of rotation during a single rotational episode. For each continuous rotational episode, a cumulative angle was calculated and 0.95 quantiles of per-fly distributions were pooled (n = 8). B) The maximal angular velocity of rotations. For each continuous rotational episode a maximal angular velocity was determined and 0.95 quantiles of per-fly distributions were pooled (n = 8). The box plots represent medians and lower and upper quartiles. The whiskers extending vertically from the boxes indicate variability outside the upper and lower quartiles, and the plus signs represent individual outlier points (For_a general reference, in normal flies median translational velocity is 30 mm/sec, with alcohol 20 mm/sec and with cocaine stage II 15 mm/sec).

As claimed previously [31], cocaine-induced behavior consists of 5 well defined stages with distinct transitions between them. Since we focus in the present study on the coordination between translation and rotation within movement segments, algorithmic division of the session into 2 stages both replicates 2 of the previously defined 5 stages and provided us with two distinct cocaine “states”, that of pre-circling (Cocaine I), and circling (Cocaine II). Division into 2 states was sufficient for fulfilling our objective of analyzing states with distinct dynamics of the angular interval. For the division we used two criteria: the cumulative percentage of three rotational modes within a movement segment, *p_rot_*, and the maximal cumulative body turn within one rotational episode, Θ. Based on the density plot of these parameters (Figures 3 and 4A), we set the thresholds as follow: *p_rot_*  = 0.85 and Θ = 360°. When at least one of the criteria was fulfilled, i.e. either *p_rot_*>0.85 or Θ_max_>360° the movement segment was assigned to the ‘circling’ stage.

#### Symbolic representation of locomotion

**Figure 5:**
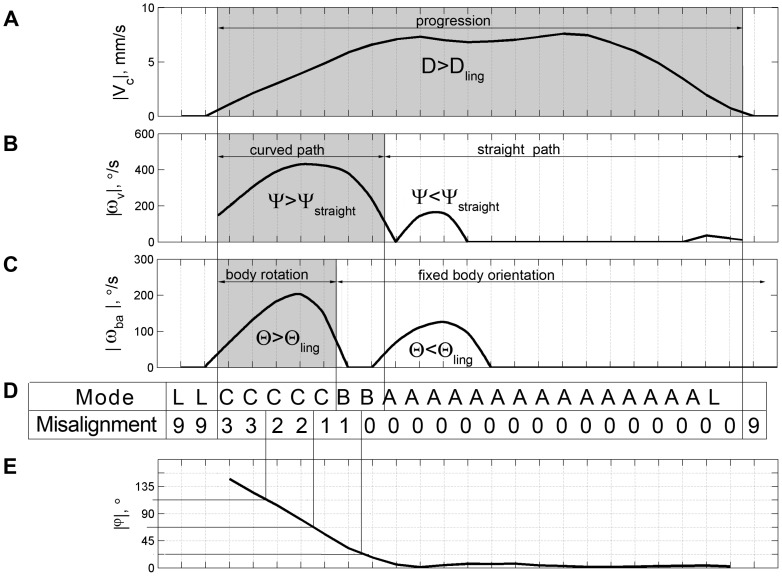
Frame classification and coding scheme. A) a plot of the scalar values V_c_ obtained during a movement segment. Progression is marked by spatial Spread, ‘D’, values exceeding the threshold distinguishing lingering (staying in place) episodes from movement segments (D_ling_ denotes the threshold value for lingering; see methods). B) A plot of the velocity vector ω_v_, denoting the change in the direction of translation of the fly's center, includes 3 bouts of change of direction, where only the first exceeds the threshold Ψ, the angular spread of the velocity vector distinguishing between straight and curved paths. C) The first 2 bouts are accompanied by a rotation of the fly, but only the first rotation exceeds the threshold of Θ, the angular spread of the fly's body orientation distinguishing between rotations and fixed orientation. The gray rectangles highlight segments of scalar and vectorial values exceeding the respective thresholds thus delineating segments marked by significant amounts of translation, curvature and rotation. The mode of coordination between the 3 kinematic variables is summarized and coded in D) by the letters: L for Lingering, C for Fixated-Front-on-Curved-Path, B for Rotation-on-Straight-Path and A for Fixed-Front-on-Straight-Path. E) The body-related directions of movement (angular interval between the direction of progression and body orientation. ±45° coded by 1; ±90° coded by 2; ±135° coded by 3; 180° coded by 4; Lingering coded by 9).

In line with our classification to modes (Fig. 5) we coded every frame by a letter (A, B, C, D, L or R) indicating its classification to one of the modes (Table 1, Figure 5D) and a number (from 0 to 4) indicating the body-related direction of progression specific to this frame. In this way, the original time series of 4 kinematic parameters is re-synthesized into one string that characterizes the original movement flow in terms of the six modes. A symbolic representation of the movement provides a useful way to study the characteristics and sequence of mode-specific clusters, which are formed by successive frames having the same letter coding.

**Table 1:** The six elementary modes of fly locomotor behavior.

	*No Rotation*	*Rotation*
*Progression on Straight path*	Fixed-front-on-Straight-path (‘A’)	Rotation-on-Straight-Path (‘B’)
*Progression on Curved path*	Fixated-Front-on-Curved-Path (‘C’)	Rotation-on-Curved-Path (‘D’)
*No progression*	Lingering (‘L’)	Rotation-in-place (‘R’)

The modes reflect the dynamics of the angular interval between the animal's direction of progression and the direction it faces.

### Pattern matching

In this study we used standard regular expression operators to draw out episodes containing a given pattern. Note that by using this procedure, we do not wish to imply that the continuous dynamic behavior can be reduced into discrete modes with hard boundaries. The procedure is merely a tool by which we simplify subsequent analysis and examine the approximate composition of the overall behavior. Several examples of regular expression patterns in standard Unix syntax are listed below:

- Mode specific clusters: ‘(m[0–4]){3,}’ (where ‘m’ was substituted by a mode specific letter). For analysis of misalignment angles within a mode, the matched sequences were further analyzed on angle categories distribution.
- Rotation around the hind legs superimposed on backward/sideways progression: ‘([BD][2]–[4])*’;
- Rotation around the hind legs was superimposed on a diagonally forward translation: ‘([BD]1)*’
- Only fixed orientation during a phase: ‘∧[AC]$’;
- Initial backward/sideward shift during start: ‘∧[AC][2]–[4]{3,}’.

### The dynamics of mode sequencing

To analyze the dynamics of transitions between clusters of frames belonging to the same mode we applied state transition analysis. The sequence of transitions between clusters was used to construct a 6×6 transition matrix A  =  (t_ij_), where t_ij_ is the number of times a cluster of mode *i* is followed by a cluster of mode *j*. Then, each value t_ij_ was normalized to the total number of changes of clusters *j* (sum_i_(t_ij_)), thereby producing a probability matrix of transition from cluster *i* to the others. This analysis was carried out only on midsections because of the relative stationarity of the movement within this phase.

### Statistical Methods

Pairwise comparisons were done either by (i) ANOVA tests and followed by Tukey's method (Tukey-Kramer when needed), or (ii) Kruskal Wallis tests followed by Wilcoxon non-parametric rank-sum tests, adjusted using the Benjamini-Hochberg False Discovery Rate (FDR) controlling procedure [34]. The choice between the two depended on the closeness to Normality of the relevant distribution.

Differences in proportions were assessed through log-linear model, or by chi-squares tests for proportion again adjusted to offer FDR≤0.05. Generalized Linear Model was used for the joint analysis of repeated measurements on same flies. All analyses were done in SPSS. Data are freely available upon request from ilan99@tau.ac.il.

## Results

### Analysis of basic kinematic variables in wild-type flies uncovers six elementary modes of motion

The method of segmentation of behavior is illustrated in Figure 5. To unambiguously describe the fly's position, we used two independent measures: the coordinates of the fly's center of mass on a fixed-frame Cartesian plane and the fly's body orientation relative to the axes of this frame, both of which are determined from video-tracking with FTrack (see Materials and Methods). Examination of the resulting time series thus allowed us to describe the fly's behavior in terms of *translation*-*related* variables (speed V_c_, direction of progression α, and changes in direction of progression ω_v_), and *rotation-related* variables (angular velocity ω_ba_, and the direction of changes in body orientation β).

Fly locomotor behavior on a substrate consists of movement segments and of staying-in-place episodes (see materials and methods). During motion, the fly either progresses over relatively large distances or performs relatively large rotations, or both. Staying-in-place episodes involve complete arrests as well as small displacements and small rotations. We term the staying-in-place segments *lingering* episodes [35]. Because lingering takes place within a circumscribed neighborhood, the spatial spread in the motion of the fly's center and the spread of the fly's body orientation do not exceed some thresholds (D_ling_, Θ_ling_). We estimated these thresholds from the overall distributions of the spatial and angular spreads of these values across all fly-sessions (Materials and Methods). The thresholds were then used to isolate three locomotor modes: progression with a nearly fixed body orientation (when D>D_ling_ and Θ<Θ_ling_), progression accompanied by rotation (when D>D_ling_ and Θ>Θ_ling_), and rotation in place (when D<D_ling_ and Θ>Θ_ling_).

**Figure 6:**
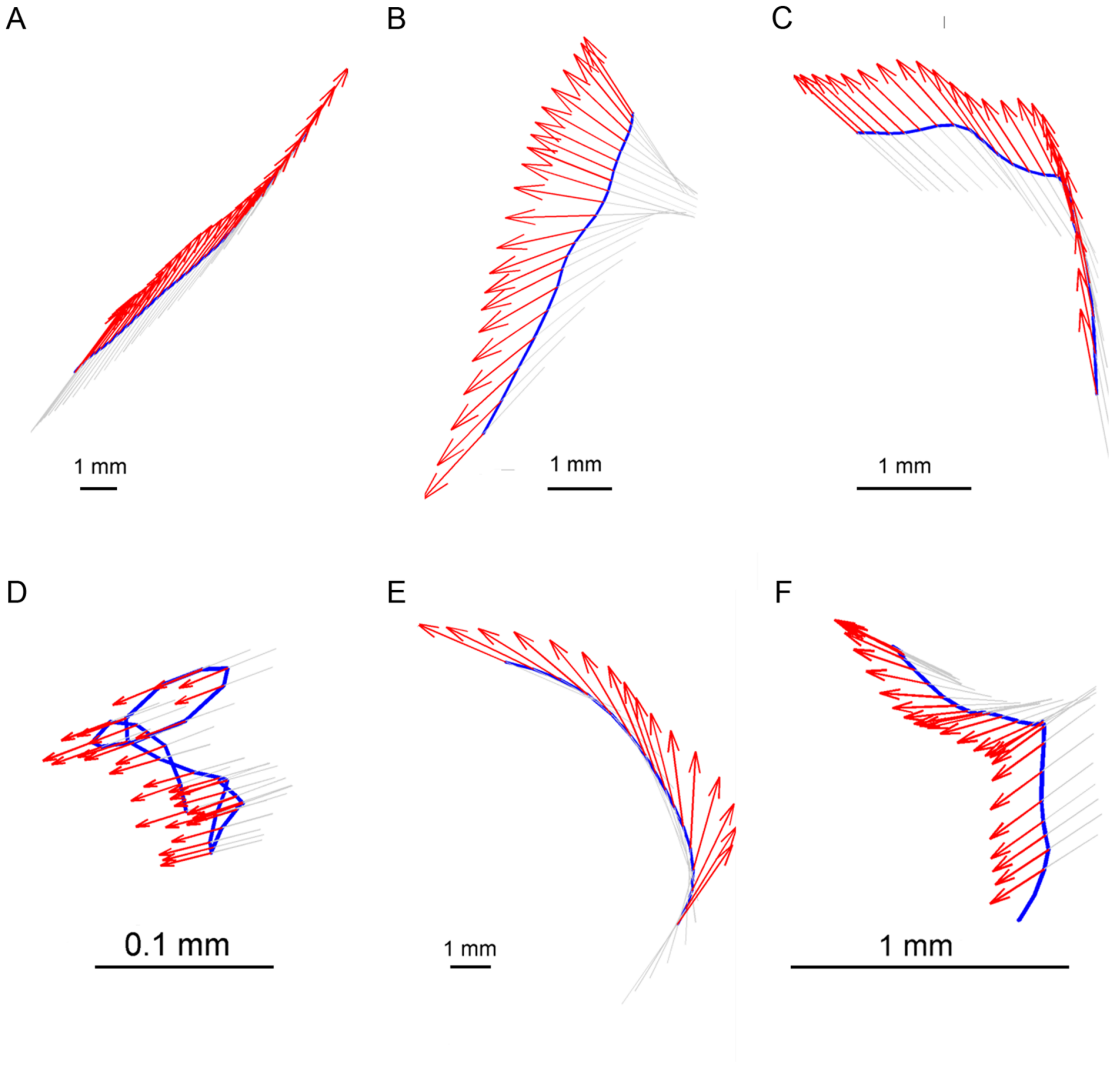
Examples of the six elementary modes of fly locomotor behavior. A) Fixed-front-on-Straight-Path (‘A’), B) Rotation-on-Straight-Path (‘B’), C) Fixated-Front-on-Curved-Path (‘C’), D) Rotation-on-Curved-Path (‘D’), E) Lingering (‘L’) and F) Rotation-in-place (‘R’). Quiver plots: blue lines represent the path traced by the mouse centre. The arrows represent the orientation of the fly's body axis.

A similar approach was used to distinguish between straight and curved segments (Materials and Methods). Even when the observer would characterize a path segment as straight, the orientation of the velocity vector slightly fluctuates; however, the angular spread of the vector (Ψ) does not exceed some threshold (Ψ_str_). Therefore, movement segments were divided into straight path segments (where Ψ<Ψ_str_), and curved path segments (where Ψ>Ψ_str_). This segmentation naturally yielded six “modes” of fly locomotor behavior, which are summarized in Table 1 and illustrated in Figure 6.

### Description of the six modes

#### Fixed-Front-on-Straight-Path

(Figure 6A; ‘A’ in Figure 5D). This is one of the two most common modes of untreated movement. Flies usually move along relatively straight paths over long distances with close alignment of the body axis and the direction of progression. Net sideward/backward translation episodes are very short in duration and path length in untreated flies (∼0.12 s and no more than 5 mm long).

#### Rotation-on-Straight-Path

(Figure 6B; ‘B’ in Figure 5D). At the start of this mode, the fly's body is typically misaligned with the direction of progression. In the course of the mode, the body rotates toward the direction of progression, typically converging to the same direction. A particular case of this mode is rotation around the hind legs, during which φ≈90° while the center of rotation is about half a body length away from the fly's center. This rotation is superimposed on forward, sideways, or backward progression, all performed along a relatively straight path traced by the center of the fly's body.

#### Fixated-Front-on-Curved-Path

(Figure 6C; ‘C’ in Figure 5D). The fly maintains a more-or-less fixed orientation while moving on a curved path. We use the term fixated rather than fixed to highlight the active, compensatory fixation, as opposed to the fixed orientation of a fly whose body axis is aligned with the direction of progression on a straight path. This mode is typically performed during a short intermediate state between Fixed-Front-on-Straight-Path and Rotation-on-Curved-Path modes.

#### Rotation-on-Curved-Path

(Figure 6D; ‘D’ in Figure 5D). In the course of this mode, the body of a fly typically rotates toward the direction of progression, the rotation and direction of displacement sign being the same. This is the second of the two most common modes in untreated fly locomotor behavior.

#### Lingering

(Figure 6E; ‘L’ in Figure 5D). Lingering episodes include at least one arrest and may also include small below-threshold displacements. Lingering duration ranges between short interruptions in movement and long (presumably sleeping) episodes.

#### Rotation-in-place

(Figure 6F; ‘R’ in Figure 5D). Rotation of the fly's body axis around a vertical axis located at the fly's body center is mostly performed in untreated flies between two lingering episodes.

Clearly, a full description of behavior must take into consideration how the alignment of the body axis is coordinated with progression. To examine the coordination between the translational and rotational variables in each mode, the relationship between the fly's direction of progression and its body orientation were described in terms of the misalignment, or angular difference between the direction the animal's center shifts, and the direction the animal faces (angular interval; φ). This angular interval can be represented as a continuous variable or be digitized into discrete angular amplitudes. Using the second option we digitized misalignment at a 45° resolution thus distinguishing 8 angular intervals of body-related directions of progression, which were collapsed into 5, by not distinguishing right from left differences: progressing forward while facing forward (φ = 0°; coded by 0), progressing at a diagonally forward (±45°; coded by 1) direction away from the direction of facing, progressing sideways (±90°; coded by 2), at right angles away from the direction of facing, progressing diagonally backwards (±135°; coded by 3), and progressing at a (180°; coded by 4) angular interval away from the direction of facing (progressing backwards) (see Figure 5D, E). As illustrated in Figure 6, the angular interval is generated by the direction of progression vector moving away from the direction of facing (front). The angular interval is typically reduced or nullified by the tendency of the front vector to rotate and align with the direction of progression (Videos S1, S2).

### A fly's use of the six modes is dynamic

**Figure 7:**
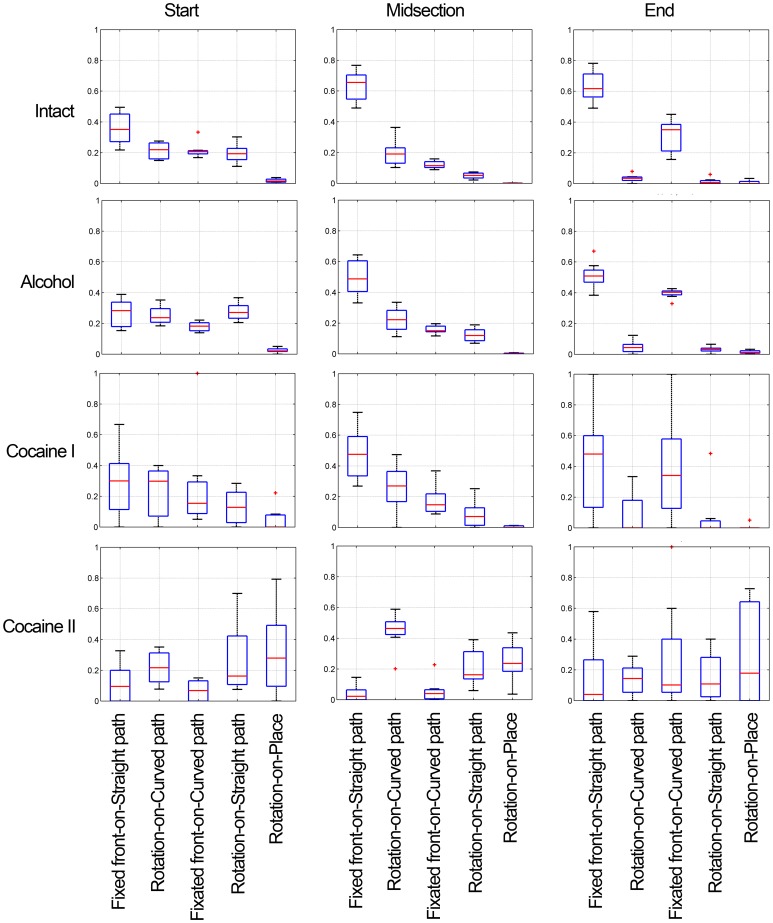
Box plot summaries of the proportion of modes used during the different phases of a movement segment in normal, alcohol- and cocaine treated flies. The box plots represent medians and lower and upper quartiles. The whiskers extending vertically from the boxes indicate variability outside the upper and lower quartiles, and the plus signs represent individual outlier points. The modes are arranged in descending order of proportion in the midsection of normal flies' panel, and this order is maintained in order to facilitate comparison in all the other panels.

Having established that fly locomotor behavior is composed of six fundamental modes, we next examined the temporal characteristics of mode usage and their coordination as a function of time. We approximated the dynamics of the process by segmenting the time of movement segments into a start, a midsection, and an end. A start extends from the initiation of movement until speed reaches half of its maximum within that segment. A midsection extends from the end of a start until speed falls down for the last time within that segment to half of its maximal value. An end consists of the remaining part of the segment. We calculated the proportion of mode usage in each temporal phase, which gives a general overview for mode usage in untreated, alcohol- and cocaine treated flies (Figure 7).

#### Midsections in untreated flies

**Figure 8:**
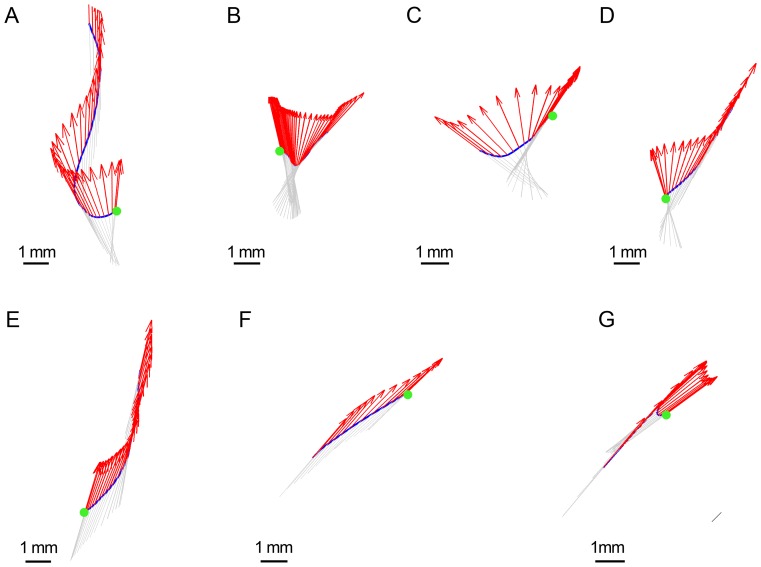
Examples of segments. A)-E) Start segments; F)-G) End segments. Red arrows indicate the fly's position for every single frame during a segment. Blue line represents the trajectory of the fly's centre, the green dot indicates the fly's initial position on Starts and the black dot indicates the final position on Ends.

The behavior of an untreated fly primarily consists of walking on a straight path with a fixed front fully aligned with the direction of progression; however, progression rarely begins with the fly's body fully aligned with the direction of progression (Figure 8). As progression continues into midsections, we see a gradual alignment of the body with the direction of progression. Rotating onto curved (13%) or straight (5%) paths toward the direction of progression, the fly's misalignment ranged between 45±22.5° to both sides. Once alignment with the direction of progression took place, however, it was maintained without fluctuations until the transition to a different mode. Thus, the midsection of progression is characterized by the existence of a stable mode with the same two transients leading into it and out of it. The stable mode was progression with a fixed (and fully aligned) front on a straight path (Figure 6A). The two transients were Rotation-on-Curved-path and Fixated-front-on-Curved-path. The transition from a straight path to a curved path involved a delay in recruitment of the body orientation to the new direction, and the establishment of a new straight path also involved a delay in full alignment with the newly established direction. Thus, a temporary misalignment with the direction of progression leads directly out of and directly into the stable mode. Sometimes, however, during the transition out of the straight line, the fly traced a curved path while maintaining its front fixated on the original direction, and only later proceeded with a rotation on the curved path (that eventually lead again to the stable fixed front on straight path). This behavior is, for example, seen when a fly walks toward a wall, and then progresses away from it while still orienting toward the wall, as if attending to the wall while its legs already carry it away from it (the sequences of mode usage in midsections were determined using a pattern matching procedure with a state transition analysis).

#### Starts in untreated flies

As the fly begins its motion, rotation toward alignment with the direction of progression occurs about two-thirds of the time, and half of these rotations (35%) occur around the hind legs. This rotation is sometimes preceded (10%) and sometimes performed simultaneously with a backward and/or sideways progression (Figure 8B). At other times the rotation is superimposed on a sideways or diagonally forward translation (Figure 8C,D). The remaining third of starts in normal flies (34%), however, do not include a rotation. In these, the fly either accelerated straight forward from its resting position (24%) or shifted its weight backward and/or sideways before proceeding straight forward (27%) (Figure 8E).

#### Ends in untreated flies

Progression segments typically ended with a rapid deceleration (∼0.3 s) while keeping the body highly aligned with the overall direction of the path (Figure 8F), except for a slight shift sideways before the final stop observed in a fifth of the cases (Figure 8G). In contrast to starts, ends rarely (3%) included rotations (p≤0.03 for all rotations adjusted for FDR). As shown, in untreated flies midsections, the fixed-on-straight path prevails, and modes involving rotation and fixations of orientation on a curved path are much less common (top mid-panel). In starts (top left panel) there is a reduction of the fixed orientation on straight path mode and an augmentation of all the other modes except rotation on curved, including fixation on a curved path (all p-values ≤0.001 adjusted for FDR). In ends (right panel) it is the fixed-on-curved orientation that is augmented and the rotation-involving modes of progression that are diminished (p≤0.03, adjusted for FDR). Flies thus tend to start a movement segment with a rotation and tend to end it with a fixation of body orientation (Figure 8).

### Drug-induced changes in the usage, sequencing, and coordination of modes

Current knowledge about alcohol- and cocaine-induced behavior in Drosophila is based on visual scoring of categories of behavior defined *ad hoc*, and on the analysis of the flies' path. Thus McClung and Hirsh [31], [36] report a transition from locomotion to circling stereotypies under cocaine, followed by a reversed sequence during recovery. With alcohol, flies were reported to display hyper locomotion and increased path curvature culminated by the performance of tight circles [37]–[39]. We examined alcohol- and cocaine-induced behavior to see if our modes could still be discerned in these preparations, to then use them to describe the overall effect of these drugs on behavior, and to examine whether the 4 respective states (1 intact and 3 drug-induced) represent distinct dynamics of the coordination between translation and rotation.

### Proportion of mode usage

#### Midsections

Alcohol increases the proportion of curved paths, of rotations on both curved and straight paths, of rotation in place, and of fixation of front on curved paths (which also involves a rotation, but in the opposite direction to that of the curving path; Figure 7). Note, that alcohol reduces greatly the proportion of straight paths with a fixed orientation, but increases the proportion of straight paths with rotations (p≤0.001 for the latter, adjusted for FDR). In other words, the 20% reduction in fixed orientation on straight path is partly due to an increase in the proportion of straight paths involving a simultaneous rotation. All in all, alcohol increases the proportion of curved paths and increases the proportion of rotations.

Partitioning of cocaine-induced behavior into two distinct modes based on intrinsic statistical and geometrical properties of the behavior (Figures 3,4,6) reveals that Cocaine stage I increases the proportion of curved paths and of rotations on the curved paths even further, and Cocaine II increases dramatically the proportion of curved paths, of rotation on curved paths, on straight paths and in place. Fixation on curved path is reduced in cocaine II compared to its proportion in cocaine I (p≤0.005 adjusted for FDR).

In summary, during midsections there is a gradual decrease in the proportion of straight paths from normal to alcohol to cocaine, a gradual *decrease* of fixed front on straight path accompanied by a gradual *increase* of straight paths with rotation; a gradual increase in the proportion of curved paths consisting of an increase in curved paths with rotations and an increase in curved paths with fixations only with alcohol and cocaine I (but not in cocaine II); and a gradual increase in rotations on curved and straight paths, and in place.

Untreated, alcohol and cocaine I repertoires thus differ in the proportions of modes from mostly performing forward progression on straight line, to mostly performing rotation. With alcohol, modes of Fixed-front-on-Straight-path are shorter compared to untreated while clusters of Rotation-on-Straight-path are longer. This trend is culminated with cocaine II, where the two fixed front modes are drastically reduced and the rotational modes are drastically increased (Figure 7, middle column of panels).

#### Starts

Alcohol enhanced the rotations on straight and curved paths correspondingly reducing the fixed orientation on straight and curved path (p≤0.005 for the first three, adjusted for FDR). Cocaine reduced fixed on both straight and curved path (p≤0.008) and increased the proportion of rotations in place.

#### Ends

Alcohol enhanced the features that characterized untreated ends by restricting the variability of the fixed and fixated modes; these, now stereotyped ends, amounted to 94% of all ends. Cocaine I increased the rotational modes, and cocaine II further increased the rotations (p≤0.014) and reduced the fixed and fixated modes (p≤0.004 for the Fixed mode, all adjusted for FDR) (Figure 7, right column of panels). As expected, treatment with cocaine causes a more pronounced decrease in the proportion of modes characterized by a fixed body orientation. This decrease was much more pronounced for ends (by ∼30% for Fixed-front-on-Straight-path and by ∼18% for Fixated-front-on-Curved-path) than for starts (by ∼5% for each). The most drastic influence of cocaine was on Rotation-in-place; the time spent in this mode increased from negligibly small, characterizing normal behavior, to 5% in starts and 17% in ends. Under cocaine treatment, variability between flies was much higher than in untreated and alcohol treated flies; it was contributed mostly by one fly who circled intensively.

### Alignment between the direction of progression and body orientation (angular interval)

**Figure 9:**
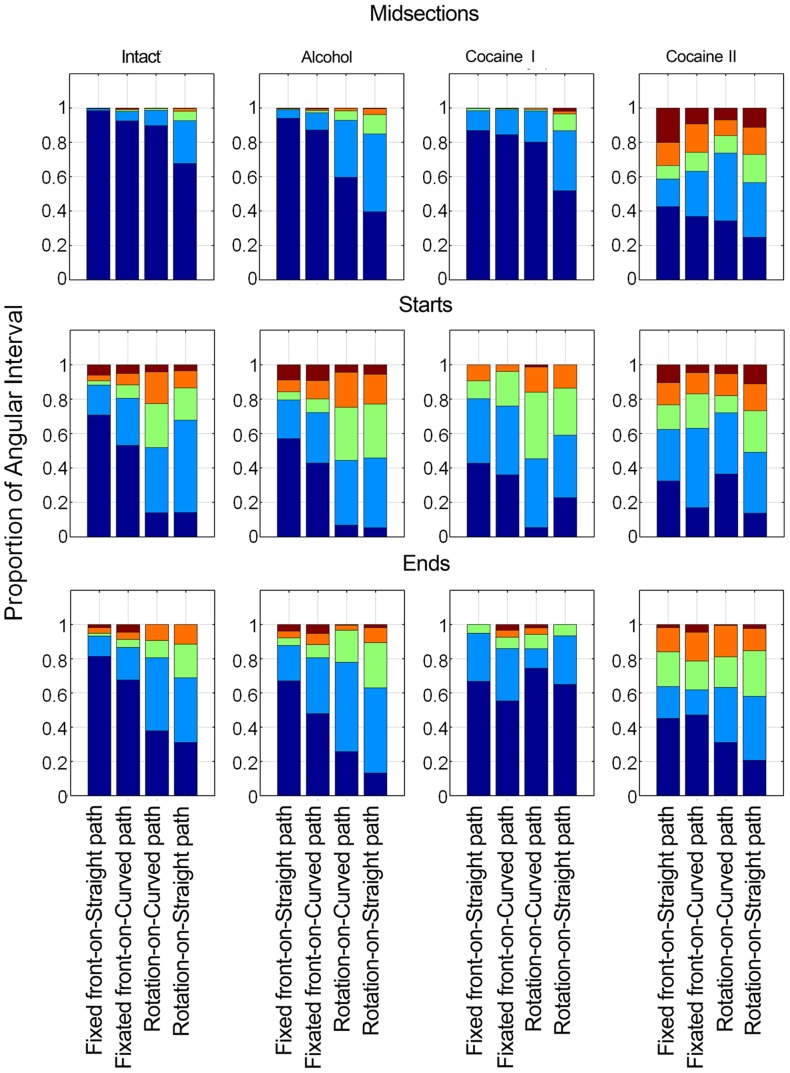
The proportion of the angular interval during the different phases of a movement segment in normal, alcohol- and cocaine treated flies. Dark blue – φ = 0±22.5; light blue – φ = 45±22.5; green – φ = 90±22.5 deg; orange – φ = 135±22.5 deg; brown – φ = 180±22.5 deg. Each stack bar represents the proportion out of the whole population of the misalignment angles characterizing each of the mode types in the normal and in the treated flies. Proportions were calculated for the data pooled from 8 flies.

Before even looking at the effect of the drugs on alignment, it is of interest to examine the effect on the angular interval of starting and ending a movement segment (Figure 9 left column). During midsections, in the absence of constraints imposed by starting or stopping, alignment was high, showing a gradual decrease from fixed, to fixated, to rotation-on-curved path to rotation on straight path. Starting had a profound effect on the angular interval, involving backward, diagonally backward, sideways, and diagonally forward progression, with the most pronounced effect seen during the rotational modes. Slowing down and stopping again involved considerable misalignment in all modes, with an increase from the fixed, through fixated, to rotation on curved path, with the highest misalignment seen in the rotation on straight path mode. The flies rotated and backed simultaneously during starts but not during ends.

#### Midsections

In Figure 9, top left panel, the modes are ordered in the untreated flies midsections in accordance to the proportion of the angular intervals involved, along an augmentation tendency from left to right.

In normal starts and ends (Figure 9, compare top left to middle left panel) the angular interval is larger than in the midsection in all modes (p≤0.001 for starts and p≤0.01 for ends), but especially in the two rotational modes. Note that the angular interval is larger in all modes, regardless of whether their proportion was increased or decreased (in Figure 7). A pronounced and sometimes very large angular interval characterizes not only the rotational modes but also the fixation of front mode. Backward walking (φ = 180; brown colored section of bar) is present in the rotational modes in starts, but not in ends.

As shown, alcohol increases the interval in most modes and time sections even if the increase is statistically significant only for Rotation-on-straight in the start and both rotations in the midsections (after adjusting for multiplicity using FDR). In spite of these changes, the ordering of the relative proportions as observed in the untreated animals is preserved throughout the treatments. With cocaine II the fixed and fixated modes are rare (see Figure 7), but if performed they involve a very large angular interval. Walking backwards, diagonally forward and backward (φ = 135; orange), and sideways (φ = 90; green), which are rare in the normal, and less rare in the rotational modes with alcohol, are common with cocaine II, accounting together for more than half of the midsection segments duration (all differences are statistically significant at the midsection, as well as fixed on straight in the start, after adjusting for FDR).

Examination of the effects of treatment within modes (comparison of first column on left in leftmost panel to first column on left in second panel on same horizontal line, etc.,) reveals an augmentation tendency across treatments: The angular interval increases in the fixed mode from untreated to alcohol to cocaine I to cocaine II, the last one involving a very big change (first left columns in panels). The same regularity applies to all the modes, even if at variable strengths and significance.

### The dynamics of mode sequencing

Movement segments consist of locomotor modes that flow smoothly from one locomotor mode into the next: the movement segment is thus continuous; the partitioning of the segment into modes is an abstraction based on the geometry of the movement, as when a rotation in place flows smoothly into rotation on a curved path which flows smoothly into rotation on a straight line. Sequencing analysis (see methods section) was carried out only on midsections because of the relative stationarity of the movement within this phase.

#### Intact

**Figure 10:**
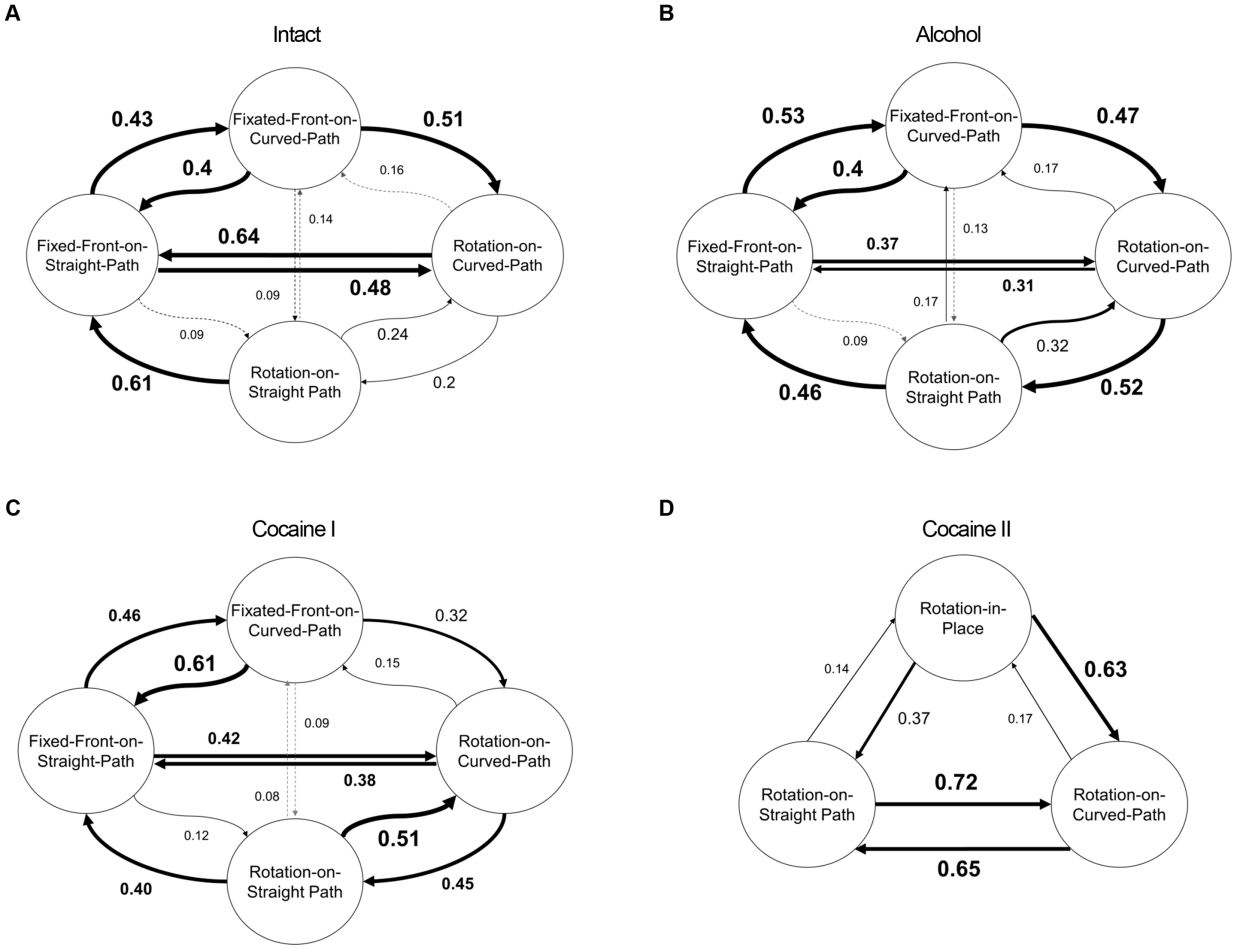
State transition diagrams for the modes during midsections in normal, alcohol-, cocaine I-, and Cocaine II-treated flies. Bold large font size numerals highlight high transition rates.

(Figure 10 top left): All modes tend to flow into a Fixed-Front-on-Straight-Path mode. Transition from this predominant mode into the second most preferred mode, Rotation-on-Curved-Path, occurs either directly or via Fixated-Front-on-Curved-Path. Transitions back from Rotation-on-Curved-Path to Fixed-Front-on-Straight-Path proceed directly.

#### Alcohol

(Figure 10 top right): Whereas intact flies shift directly from Rotation-on-Curved-Path to Fixed-Front-on-Straight-Path, alcohol-treated flies tend to do it via Rotation-on-Straight Path. Following a transition from progression on a curved path to progression on a straight path, alcohol-treated flies show a higher than intact delay in the alignment of their body axis with the direction of the straight path.

#### Cocaine I

(Figure 10 bottom left): At the beginning of cocaine action the transitions between modes are more similar to those observed with alcohol, rather than with intact flies.

#### Cocaine II

(Figure 10 bottom right): The very low probability of switching to Rotation-in-Place reflects the fact that in 43% of the movement segments performed during this stage of drug action midsections are composed entirely of this mode. When not in this mode the flies alternate between Rotation-on-Curved-Path and Rotation-on-Straight Path (0.72 and 0.68) rarely shifting to Rotation-in-Place (0.14 and 0.17). After Rotation-in-Place, the flies tend to switch to Rotation-on-Curved-Path (0.63) rather than to Rotation-on-Straight-Path (0.37).

## Discussion

The main findings and biological insight provided by the present study is that i) the angular interval between the direction a fly walks and the direction it faces is actively managed by the fly's Central Nervous System, and ii) the amplitude of this interval is increased with alcohol and increased much further with cocaine II. This type of effect has not been reported for any drug, let alone alcohol and cocaine. Furthermore, a comprehensive analysis of the coordination between translation and rotation, the two degrees of freedom that exhaust the behavior of any bilateral organism at the scale of the path has, to our knowledge, never been performed systematically on any organism, let alone fruit-flies and in a high content fashion. The way in which a fly first sets its direction of translation, followed by a fast (in intact flies) or slow (with cocaine) convergence of its body orientation to the direction of its translation is illustrated in videos S1 and S2.

Before discussing how the angular interval and other kinematic aspects of locomotor behavior are actively managed in the tested states we put our methodology into historical context and validate this type of analysis.

The tools that are available at the time of a study determine the time scale and the spatial scope of the studied phenomenon. Until recently, tracking and data-storing technology restricted neuroethological studies to short-duration spatially restricted behaviors. Prey capture in the praying mantis [40], prey-capture flight in dragonflies [41], negotiation of barriers and adaptation to slippery ground in cockroaches [42], fixating objects in the face of expanding optic flow [43] or turning in fruit flies [44] were studied because their narrow spatiotemporal scale allowed tracking of low-level kinematic variables such as trunk and head orientation, direction of stepping and body translation and moment-to-moment velocity, all necessary for the study of the CNS/behavior interface but also accessible technologically. The fine resolution data in these studies further allowed dynamic representations, which could then be juxtaposed vis-à-vis concurrent myographic and neural variables. In contrast, tracking and data-storing technologies were too limited for recording behavior in large arenas for long intervals. Since pharmacological and genetic studies required the recording of behavior at these scales, measures were taken in the aggregate for large parts of the session, such as distance travelled or percent time in center [45], [46], or path curvature [39] or the scoring of expert-determined patterns such as “circling”, “rotating” and “backward walking” [31], [36], or the drawing of selected portions of the path [13], [38]. As subjective as these patterns were, and as unarticulated these drawings were, they proved indispensible for comparing closely related preparations. Using them as replacement for kinematic variables was, however, a mixed blessing. For example, adhering to behavior as a sequence of *ad hoc* “patterns” or “response categories” is tricky: in the majority of cases, these alleged patterns can not be shown to have a neural reality; they are used as black boxes whose kinematic content is disregarded; they impede comparisons across dissimilar preparations and species; and they do not allow the study of coordination. For example, *a priori* definitions of “patterns” like “circling” and “rotation” in fly cocaine-induced behavior bars the observation that a gradual reduction in translation transforms circling into rotation-in-place [47], and an *a priori* “backward progression” category obscures the dynamic context in which this and a whole range of other intervals ranging between 0° and 180° are lawfully embedded.

Current developments in tracking and computational technology beg for a shift toward a high content phenotyping based on data driven quantifiable dynamics. While such approach has been implemented in few studies [21]–[29], a current common trend has been to train automatic pattern detectors to use the high-quality data to reinstate the intuitive patterns that made quantitative ethology inadequate for comparisons across species and taxa in the first round [48], [49] and now threaten to slow down behavioral Neuroscience in the second round [20].

What signifies the present study is that it not only tracks kinematic variables continuously, but also adheres to a dynamic representation of their coordination all the way to the final descriptive model. In other words, both initial and final results are formulated in dynamic terms. Building blocks are, furthermore, defined on the basis of their intrinsic dynamics, so that constructs like the 6 coordinative modes constitute end products rather than patterns established by connoisseurs.

The coordination between an untreated fly's shift of weight (translation) and its shift of front (rotation) is illustrated in video S1. In this animation we use a mode of presentation that is complementary to the quiver plots used so far (Figures 6,8): here, instead of tracing the path generated by the progression of the fly's center of mass, we represent the velocity vector, whose direction and length indicate the momentary direction and speed of shift of weight of the fly. The fly's front is indicated by the thick red line. As shown, the fly first shifts weight in a new direction and only then, with a small delay, shifts front so as to align with the new direction of progression. It is the coordination between these two vectors that is the subject of the present study. As shown, in untreated flies the interval between the two vectors is small, short lived, and annulled as soon as the fly's orientation converges to the direction of progression. It is as though the “gain”, transforming the input signal generated by the change in the direction of progression is high, resulting in an almost immediate converging response of the front system (fast chasing of the front vector after the velocity vector). In contrast, with cocaine (video S2), this “gain” is much lower: large angular intervals are closed gradually over a long time interval following a large change in the direction of progression and a much higher speed (slow chasing). In untreated flies the angular interval is small and brief, with alcohol it is increased and more extended in time, and with cocaine it is greatly increased and greatly extended.

The differences between intact, alcohol- and cocaine-treated behavior include, respectively, a decrease and a substantial decrease in i) “gain” (amplitude of the angular interval and the latency to close it) and an increase and a substantial increase in ii) the proportion of curved paths, and in iii) the proportion of rotational modes involving shift of front (Figure 3). In addition, there is, with alcohol and with cocaine I, an *iv) increase* in the proportion of fixation on curved paths. Whereas in normal flies the fixed front on straight path is characterized by a small angular interval, with alcohol and much more so with cocaine the fixed mode on straight path may involve a large angular interval of up to 180 degrees (Figure 3 top right panel). The proliferation of segments involving a fixed front with a large angular interval on straight path, and fixations of front on curved paths implies that “gain” magnitude alone does not account for the observed differences between intact and drugged behavior: additional constraints are required to maintain a fixed/fixated front in a fly being oriented one way and proceeding the other way, on a curved or straight path. Thus, the increased proportion of curved paths and the lower gain do not account fully for the large interval, or for the increased proportion of rotational modes.

**Figure 11:**
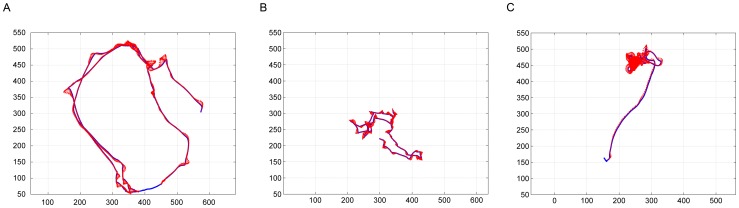
Twitter plots of selected segments of locomotor behavior presented in videos S3–S5. Continuous line presents the path traced by the fly's center. Arrows present the fly's moment-to-moment body orientation (front).). A) a sober fly; B) a fly treated with ethanol; C) a fly treated with cocaine.

Working our way up from the four features listed above, we can appreciate the “forces” that shape the fly's behavior. Intact behavior is characterized by long segments of fixed front on straight paths, absence of rotations in place, relative paucity of curved paths and of rotational modes, small angular intervals and high gain (video S3; fig. 11A).

With alcohol, behavior is characterized by shorter segments of fixed front on straight paths, more frequent rotations in place, proliferation of curved paths, wider angular intervals and lower gain creating the impression of seemingly aimless and “indecisive” behavior in the central portion of the arena, as opposed to the wall-to-wall arena-crossing behavior characterizing the intact flies (video S4; fig. 11B). Alcohol also augments the angular amplitude of single rotational episodes by increasing their duration (Fig. 4A) without increasing their angular speed (Fig. 4B). Yet, the speed of progression is reduced with alcohol, which is consistent with previous results [37]. Another feature, not observed in this clip, is the staggering gait characterizing both fly and human behavior with alcohol: sideways shifts of weight involving sideways stepping all the while fixating the direction of the human or fly's front.

With cocaine, fixed front on straight path involving zero angular intervals are almost eliminated, being replaced by straight segments involving large, fixed and fixated intervals, path curvature is gradually augmented, turning into rotation in place, all involving large angular intervals and low gain (video S5; fig. 11C).

Assignment of the frame-by-frame instantaneous movement to one of the six modes, and concatenation of the frames into clusters belonging to each of the modes partitioned the path and allowed us to quantify the behavior in terms of the proportions and dynamic sequencing of the six modes, which were defined similarly for all animals. Finally, using a universal, low-level kinematic classification system which unambiguously characterizes planar motion provided a common basis for a comparison between seemingly very different behaviors and may provide the fine measurements necessary for future high content pharmacological or genetic phenotyping studies on the one hand, and comparisons of the relationship between shift of weight and shift of front across taxa, from fruit flies to man, on the other hand.

## Supporting Information

Video S1 **The angular interval between an intact fly's direction of progression (direction of shift of weight of the fly's centre of gravity) and its orientation (the direction of the fly's front).** The front of the fly is represented by the orientation of the thick red bar. The direction and magnitude of progression (also termed the velocity vector) are represented by the thin blue line attached to the forepart of the thick bar. As shown, weight is shifted first, in a new direction, and front converges or tends to converge to the new direction established by the weight shift (which is also the direction of progression). The angular interval is generated by the direction of progression vector moving away from the direction of front. The angular interval is reduced or nullified by the tendency of the front vector to rotate and align with the direction of progression. Note the small magnitude of the angular interval and of the velocity magnitude in this intact fly (“high gain”) compared to the large magnitude of these values in the cocaine treated fly in video S2.
(MP4)

Video S2 **The angular interval between a cocaine treated fly's direction of progression (direction of shift of weight of the fly's centre of gravity) and its orientation (the direction of the fly's front).** For further explanations see video S1. Note the relatively large amplitude of the angular interval and of the velocity magnitude in this cocaine treated fly (“low gain”) compared to the corresponding values in the intact fly.
(MP4)

Video S3 **A selected segment of intact fly locomotor behavior in the circular arena.** A cursor is superimposed on the fly's video image by the tracking system. Note long segments of fixed front on straight path, absence of rotations in place, relative paucity of curved paths and of rotational modes, small angular intervals and high gain, all characterizing intact fly behavior.
(MP4)

Video S4 **A selected segment of fly alcohol induced locomotor behavior in the circular arena.** A cursor is superimposed on the fly's video image by the tracking system. Note shorter segments of fixed front on straight path, more frequent rotations in place, proliferation of curved paths, larger angular interval and lower gain all in comparison to intact fly behavior. All these features together create the impression of seemingly aimless and “indecisive” behavior in the central portion of the arena, away from walls, as opposed to the wall-to-wall arena-crossing behavior characterizing the intact flies.
(MP4)

Video S5 **A selected segment of fly cocaine induced locomotor behavior in the circular arena.** Note the almost complete elimination of paths involving fixed front with zero angular interval on straight path. These segments are replaced by similar segments involving large, fixed and fixated intervals (in the video clip the fly progresses north east while fixating its front northwards), path curvature is gradually augmented, turning into rotation in place, all involving large angular intervals and low gain.
(MP4)
